# Outcomes of secondary autologus dermo-fat orbital implants in anophthalmic sockets

**DOI:** 10.12669/pjms.37.2.3209

**Published:** 2021

**Authors:** Nausheen Hayat, Saad Jan, Natasha Atiq, Alyscia Cheema

**Affiliations:** 1 Dr. Nausheen Hayat, FCPS, MRCSEd Opth (UK), Consultant Ophthalmologist, Faculty Member, Department of Ophthalmology, Jinnah Post Graduate Medical Centre, Karachi, Pakistan; 2 Dr. Saad Jan, MBBS, MD. Post Graduate Resident, Department of Ophthalmology, Jinnah Post Graduate Medical Centre, Karachi, Pakistan; 3 Dr. Natasha Atiq Final year medical student, Bahria University Medical and Dental College, Karachi, Pakistan; 4 Professor Alyscia Cheema, FCPS, FRCSEd (UK), Head of Department, Department of Ophthalmology, Jinnah Post Graduate Medical Centre, Karachi, Pakistan

**Keywords:** Dermis fat graft, Anophthalmic socket, Orbital implants

## Abstract

**Objectives:**

To evaluate the outcomes of secondary autologous dermis-fat graft as an orbital implant in anophthalmic sockets.

**Methods:**

In this prospective study, which was conducted at Jinnah Post Graduate Medical Centre, Karachi, between January 2015 and January 2020, we evaluated 12 patients between the ages of four and 60 years. Most of the adults were victims of trauma, whereas children were known cases of retinoblastoma or trauma and all underwent enucleation. All of them were primarily treated elsewhere and not offered primary orbital implants. We performed autologous dermis-fat graft as an orbital implant in these patients harvesting graft from gluteal region and followed them up to look for complications.

**Results:**

Out of 12 patients two went into failure, while rest of the patients showed successful outcome. All patients underwent successful surgery. Initially, a silicon conformer was placed, which was later on replaced with artificial prosthetic eye.

**Conclusion:**

Regardless of the small sample size, this procedure proved to be a safe and effective method for augmenting orbital volume in anophthalmic sockets in children and adults.

## INTRODUCTION

The dermis fat graft (DFG) was first described by Smith and Petrelli in 1978^1^ for anophthalmic socket reconstruction and since then, has become a widely employed technique by which we can increase orbital volume in enucleated patients.^2^ The technique can be utilized as a primary procedure after enucleation, especially in young patients, who in particular have troublesome conditions, like inflammatory hot socket after trauma, significant conjunctival scarring and as well as a secondary procedure for chronically exposed orbital implants, empty sockets after expulsion of orbital implant and sockets that have contracted.

An Autologous DFG consists of dermis and subcutaneous fat, after removing the epidermis, and is a sustainable volume replacement implant for primary enucleation and evisceration.^3^-^6^ The dermis in the DFG is thought to improve vascularization and reduces the chances of fat atrophy. Also, it functions as a barrier, opposing fatty augmentation. In this way, the graft provides, not only a soft tissue and surface lining with vascular support, but it also functions as a temporary biologic dressing.^7^,^8^ The gluteal area is the site mostly used to harvest the graft, but regions such as the abdomen and the periumbilical can also function as a graft harvesting site.

Also, regardless of the popularity of alloplastic grafts, one should not overlook autologous grafts as an orbital implant, because complications such as exposure and extrusion have been reported from alloplastic grafts and there is lesser chance of migration or extrusion of the graft with the use of autologous tissue as compared to the use of alloplastic grafts.

Therefore, in this prospective study, we aimed to evaluate the outcomes of secondary autologous dermis fat graft as an orbital implant in anophthalmic sockets. To the best of our knowledge, no work has been done before in this regard, locally and nationally.

## METHODS

In this prospective case series, 12 patients were operated between January 2015 and January 2020 at Jinnah Post Graduate Medical Centre, Karachi, were evaluated. Approval from the ethical committee of the institute was obtained vide letter NO.F.2-81/2020-GENI/46693/JPMC (dated: 14-09-2020) and informed consent was taken from all the patients beforehand. There were 07 male and 05 female patients. All surgeries were performed by the same surgeon under general anesthesia.

The inclusion criteria consisted of all patients between the ages of four and 60 years, including both genders, who had already undergone enucleation without implant in their primary surgery, due to tumor, burns and trauma which included perforated globe, blast injuries and globe lacerations. Also included in our research were patients who had long term exposed implants, implant extrusion or contracted sockets. The exclusion criteria consisted of patients younger than four years and older than 60 years of age, patients under radiotherapy or having any inflammatory disease of the orbit, and unwilling patients.

### Surgical Procedure

After obtaining informed written consent, preoperative evaluation for general anesthesia was performed for all the patients.

**Fig.1:**
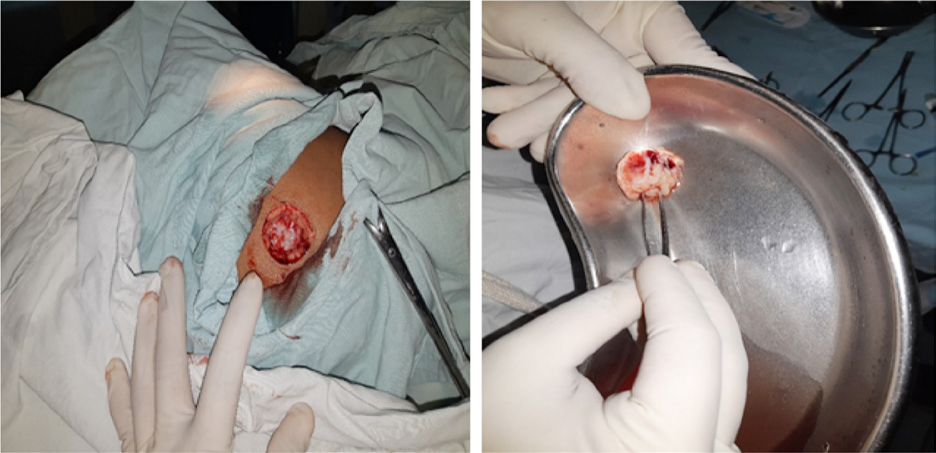
Showing DFG harvest site after removal of the graft on t he left and the removed DFG on the right, that is ready for implantation.

This is a two-step procedure, the first step is to harvest the graft, for which, the patient is placed in a lateral position on his contralateral hip and the donor site which lies in the upper outer quadrant of the ipsilateral hip is marked. After marking the donor site which lies 5 cm superior of the middle point between the anterior superior iliac crest and ischial tuberosity of the femur, an elliptical shape with a diameter of 2.5 cm was created. The donor site was injected with 2% Xylocaine with adrenaline for adequate hemostasis. An elliptical or crescent shaped incision was given of required size and the epidermis was carefully removed without damaging the underlying dermis. A deep incision of about 2 to 3 cm deep was given through the dermis and subcutaneous fat, avoiding fascia and DFG of approximately 2.5 cm depth was harvested [Fig.1]. The donor site is then closed in two layers with absorbable and silk sutures respectively.

**Fig.2:**
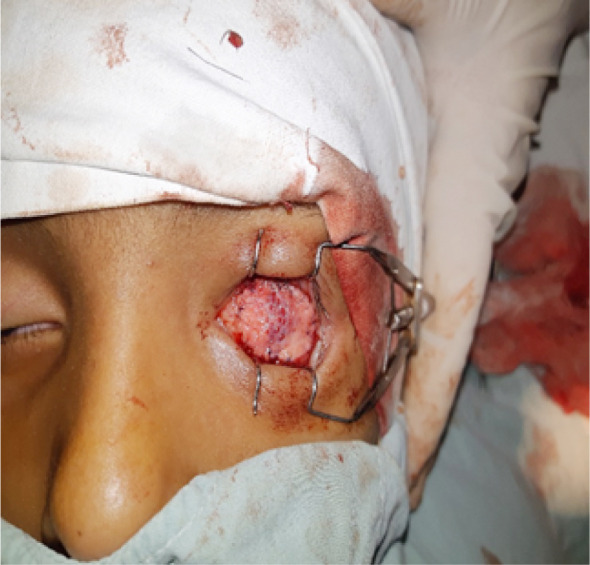
Showing DFG after implantation in the patients left orbit.

After repositioning the patient, the second step is to prepare the socket. The conjunctiva was infiltrated with 2% xylocaine and adrenaline, after which, a horizontal incision was given in the conjunctiva. Subsequently, scarred tissue was removed and adhesions were released with the help of conjunctival scissor and blunt tipped artery forceps to create space for implant. The DFG was implanted in the socket with dermis on the outer side [Fig.2]. The conjunctiva was sutured to the surrounding dermis with absorbable (vicryl) sutures. A conformer was then placed for protection. No per-operative complications occurred among any of the surgeries.

Post operatively oral antibiotics and analgesics were given along with topical antibiotics and steroids. The harvest site wound was observed and sutures were removed after 1 week. Weekly cleaning of conformer with pyodine was done in the Ophthalmology operating room at the hospital for four weeks. After four to six weeks, an artificial shell was implanted after taking the size of the graft bed [Fig.3].

**Figure:**
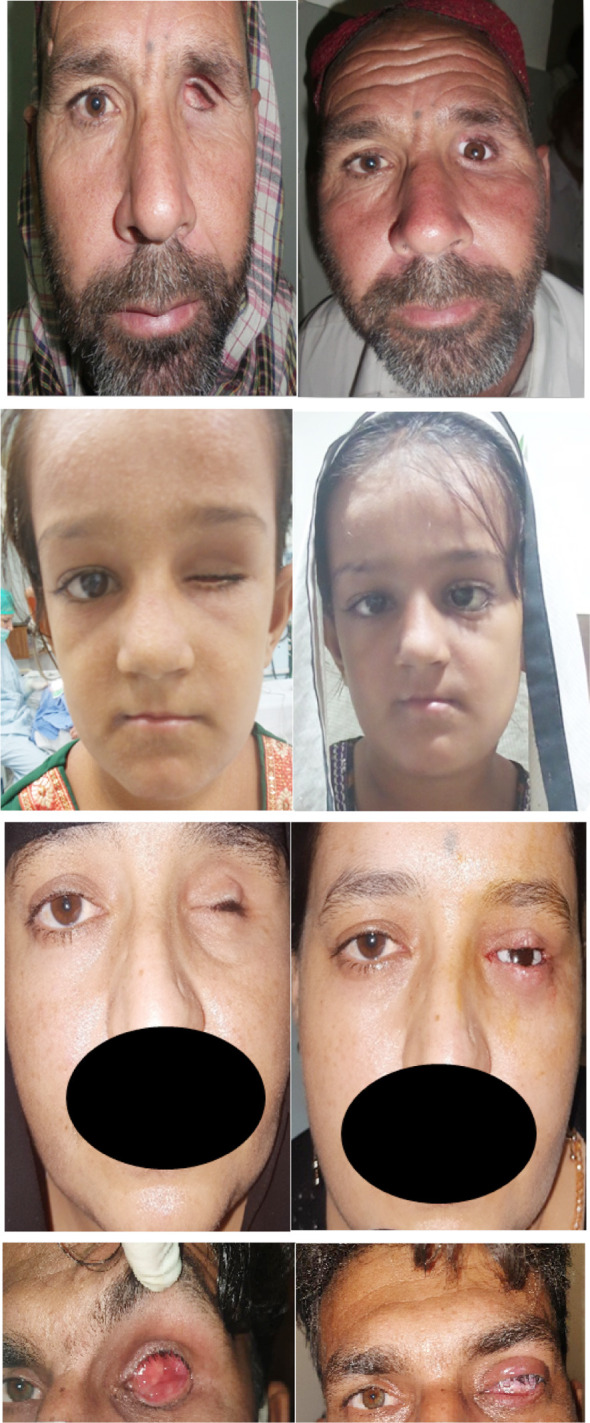
Fig. 3a, 3b, 3c and 3d showing pre and post-operative pictures of successfully implanted prosthesis following DFG.

## RESULTS

The study comprises of 12 patients, out of which, 7(58.33%) were males and 5(41.67%) were females. The mean age of the patients was 25.38±19.42 years.

The most common primary diagnosis in younger patient population (6 subjects) was retinoblastoma, whereas in the older patient (six people) population, the most common primary diagnosis was found to be trauma/globe perforation. Socket contraction came out to be the most common pre-operative diagnosis with 50% (six patients) followed by anophthalmic eye (4), while globe perforation and pthysical eye were other causes. The primary surgery in all the patients was enucleation whereas one patient also had a concomitant DFG at the time of primary surgery.

As a result, 10 (83.3%) out of 12 patients were satisfied with the surgical outcome, and reported their ebullition for results, with only two patients (16.6%) having failed surgery. One of them (patients who went into failure) was a nine year old child, had history of receiving radiotherapy for retinoblastoma before orbital implant surgery and developed fornix contraction with fat resorption after DFG, which was additionally treated by buccal mucosal graft and re-do DFG. Whereas the other patient, an adult female, who already had an unsuccessful DFG procedure performed elsewhere, after her re-do DFG by us, developed fornix scarring and conformer extrusion, which was subsequently treated by fornix reconstruction with Amniotic membrane graft as shown in Table-I.

**Table-I:** Demographic data of the patients who underwent secondary autologous Dermis Fat Implants.

*Patient No.*	*Age (yrs.)*	*Sex*	*Primary Diagnosis*	*Preoperative Diagnosis*	*Primary Surgery*	*Follow up (months)*	*Complications*	*Additional Treatment*	*Results*
1	50	M	Globe perforation/blast injury	Socket contraction	Enucleation	6	Ø	Ø	Satisfied
2	6.5	F	Retinoblastoma	Socket contraction	Enucleation	6	Ø	Ø	Satisfied
3	9	M	Retinoblastoma	Socket contraction	Enucleation	12	Fornix contraction, fat resorption	Buccal mucosa graft, re-do DFG	Failed
4	53	M	Childhood injury	Socket contraction	Enucleation	6	Ø	Ø	Satisfied
5	32	F	Burn injury	Socket and lids contraction	Enucleation, DFG	12	Fornix scarring, conformer extrusion	Fornix reconstruction with Amniotic membrane graft	Failed
6	14	F	Retinoblastoma	Socket and lids contraction	Enucleation	6	Ø	Ø	Satisfied
7	53	M	Globe perforation	Anophthalmic socket	Enucleation	6	Ø	Ø	Satisfied
8	5	F	Retinoblastoma	Anophthalmic socket	Enucleation	6	Ø	Ø	Satisfied
9	12	F	Globe perforation	Anophthalmic socket	Enucleation	6	Ø	Ø	Satisfied
10	4	M	Retinoblastoma	Anophthalmic socket	Enucleation	6	Ø	Ø	Satisfied
11	28	M	Firearm injury	Globe perforation	Enucleation	6	Ø	Ø	Satisfied
12	38	M	Childhood injury	Phthisical eye	Enucleation	6	Ø	Ø	Satisfied

## DISCUSSION

Autologous dermis fat grafts (DFG) are widely used as a technique to augment orbital volume in eviscerated or enucleated eyes, until such time that a prosthetic orbital implant could be placed in the affected eye. DFGs are used after eviscerations or enucleations, and can be implanted primarily, at the time of primary surgery, or secondarily. DFG is implanted weeks, months or in many cases, years after primary surgery, leading to severely contracted sockets, in which case socket preparation is required to remove scarred or unwanted tissue.^9^

DFG remains in the socket over the course of next few weeks, 4 to 6 weeks in our case, after which, the inflammation subsides and prosthesis can be implanted in the socket. DFG provides the advantages of being a cheaper, relatively easily available and safer alternative for orbital volume maintenance in enucleated sockets,^10^ as alloplastic materials are prone to develop toxic reactions, infections, foreign body reactions, and late exposure following conjunctival erosions. However, in recent years with the advent of newer biomaterials with improved capabilities, which include an antibacterial effect and also enhanced angiogenesis at the site,^11^ these adverse effects have been minimized. Moreover, DFGs seem to provide additional epithelial lining in patients with shrunken conjunctiva and forniceal shortening,^12^,^13^ which can be augmented by an amniotic membrane grafted over the DFG to prevent exposure of fat and assist in the healing of conjunctiva.^14^

In our study, we prospectively investigated the long term outcomes of secondary DFGs. The indications for DFG in our study include socket contraction, followed by anophthalmic eye, which portrayed a stark difference when compared to Aryasit et al.,^2^ which showed exposure (31.7%), extrusion (26.8%) and volume insufficiency with a shallow fornix (24.4%) as the most common indications. This could be because they performed DFG implant surgery on patients that already had an allogenic implant in place, whereas Karatas et al.15 had socket contraction as the most common indication for secondary DFG, which is similar to our study. The final outcome, patient satisfaction and the rate of complications were evaluated. We found out that 10 (83.3%) out of 12 of our DFG patients were satisfied with the aesthetic and functional outcome, whereas

Karatas et al.^15^ evaluated 17 patients out of which 10 patients were operated for secondary DFGs and reported 60% (6 patients) of the patients being satisfied with the results with no major complications, and four patients (40%) were not happy with outcomes.

Another study which showed less favourable outcomes in comparison to our work, was done by Nentwich et al.^16^ also evaluated 66 secondary DFG patients in their study. It was a retrospective study with a relatively larger sample size and it showed 57% of the patients being highly satisfied with their results with major complications being rare.

A recent study conducted by Fasina^17^ showed high success rate similar to our study, like in treating all nine patients with a secondary DFG, giving satisfactory cosmetic results in almost all subjects.

Complications could be seen in a subset of patients. We had two patients with complications, one of them, a child, had fornix contraction with fat resorption after DFG, which we believed was due to the history of radiotherapy for retinoblastoma. This effect of radiation, leading to fat resorption and/or fat atrophy was also seen by Karatas et al.^15^ Our one adult female patient had fornix scarring and conformer extrusion, for unexplainable reason, however the cause of loosing her eye was trauma.

Minor complications which can be seen in DFG patients and are quite common include, keratinization of the graft, hirsutism of the graft, pyogenic granulomas and conjunctival cysts, whereas major complications include, infection, ulceration or necrosis of the graft and spontaneous graft atrophy.^18^

### Limitations of the study

Although this research work has shown some promising results, however it has a few limitations, such as small data size and a short follow up period, because majority of the patients belonged to rural areas and were unable to follow-up on a regular long term basis. Moreover, small data size indicates that inspite of the fact that such cases exist in the society, however lack of awareness and knowledge about approaching the right specialist of this work, creates the delay for the treatment.

### Recommendation

We recommend that a mass level awareness programs must be carried out by the government and the civil society with mutual collaboration in regard to all kind of orbital diseases and their treatment.

## CONCLUSION

We would like to document that secondary autologous DFGs have proven to be a cheap and safe method of orbital volume replacement, as it is easily harvested from the patient’s body and has no foreign body reaction. It has shown good outcome in a vast majority of our patients with few complications.

### Authors’ Contribution:

**NH:** Conceived, designed, operated all the patients & editing of manuscript and is responsible for integrity of study.

**SJ & NA:** Did data collection, statistical analysis and manuscript writing.

**AC:** Provided general support and helped in making diagnosis.
